# Supplementary material for: A Versatile Two-Step CRISPR- and RMCE-Based Strategy for Efficient Genome Engineering in Drosophila
Source: G3 (Bethesda). 2014 Oct 15;4(12):2409–18. doi: 10.1534/g3.114.013979 (PMC4267936; doi:10.1534/g3.114.013979)
Supplement: Supporting Information [file supp_4_12_2409__index.html]

A Versatile Two-Step CRISPR- and RMCE-Based Strategy for Efficient Genome Engineering in Drosophila — Supporting Information 

# A Versatile Two-Step CRISPR- and RMCE-Based Strategy for Efficient Genome Engineering in *Drosophila*

## Supporting Information for Zhang, Koolhaas, and Schnorrer, 2014

**Files in this Data Supplement:**

- Supporting Information - Figure S1 and Table S1 (PDF, 660 KB)
- Figure S1 - PCR scheme to verify the correct HDR event. (PDF, 580 KB)
- Table S1 - All primer sequences used in this study. (PDF, 496 KB)
