# Supplementary material for: A Versatile Two-Step CRISPR- and RMCE-Based Strategy for Efficient Genome Engineering in Drosophila
Source: G3 (Bethesda). 2014 Oct 15;4(12):2409–18. doi: 10.1534/g3.114.013979 (PMC4267936; doi:10.1534/g3.114.013979)
Supplement: Supporting Information [file supp_g3.114.013979_013979SI.pdf]

G3· Genes | Genomes | Genetics

A versatile two-step CRISPR- and RMCE-based strategy for efficient genome engineering in *Drosophila*

Xu Zhang[1], Wouter H. Koolhaas[1] and Frank Schnorrer[1]

[1]Max Planck Institute of Biochemistry, Am Klopferspitz 18, 82152 Martinsried, Germany.

Correspondence:

schnorrer@biochem.mpg.de, Tel: +49 89 8578 2434

DOI: 10.1534/g3.114.013979

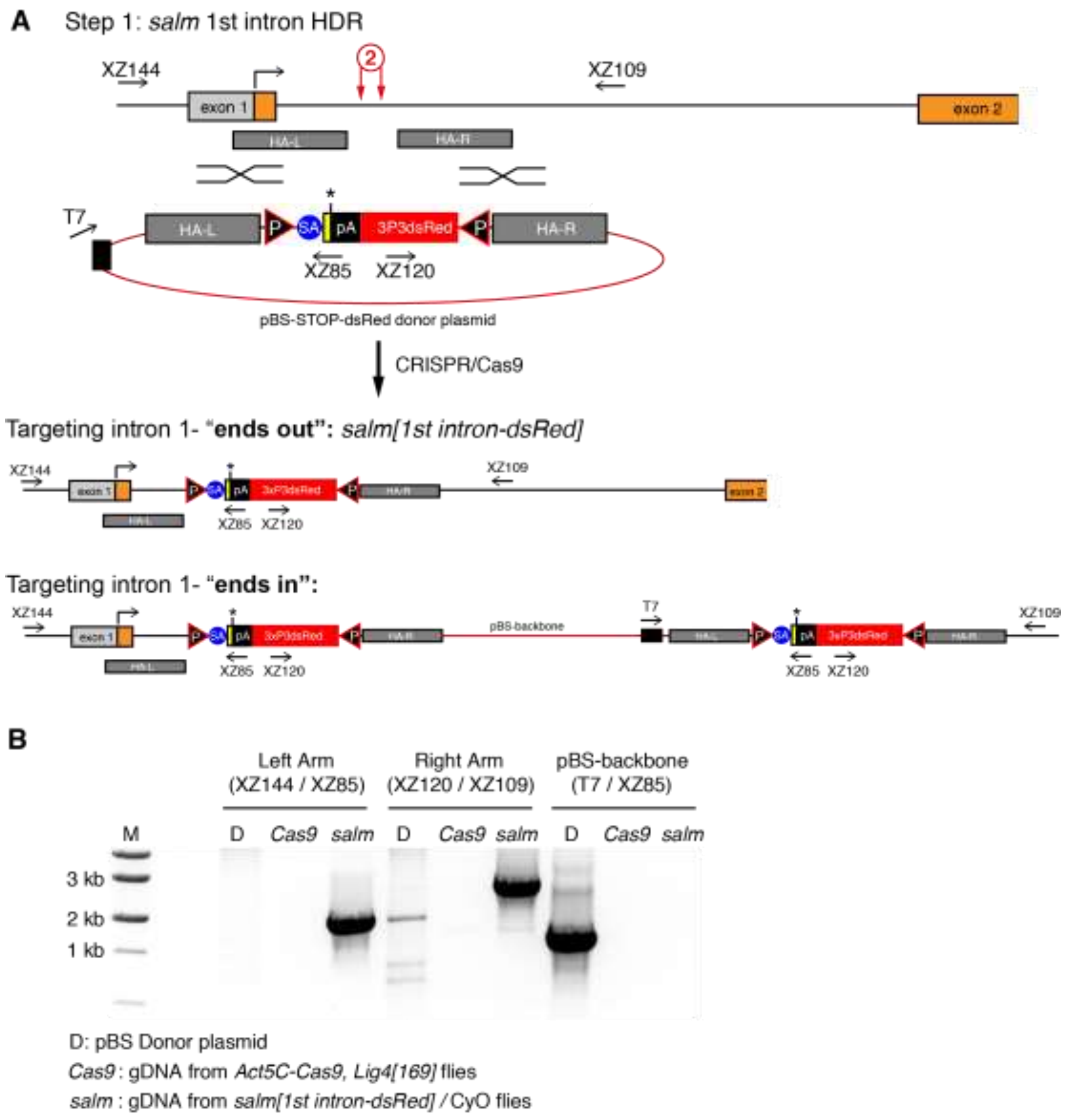

Figure S1 PCR scheme to verify the correct HDR event.

(**A**) Scheme for the generation of *salm[1st exon-dsRed]* by HDR. Possible results of "ends-out" and "ends-in" homologous recombination are shown, including the positions of the homology arms and the primers used for PCR. Note that only "ends-in" homologous recombination results in the pBS-backbone in the genome, which can be detected by PCR with primers T7 / XZ85. (**B**) PCR verification of the "ends-out" insertion of the dsRed-STOP cassette in the 1st intron of *salm*. Left and right arms amplify only from DNA isolated from the *salm[1st exon-dsRed]* flies. Primers XZ144 and XZ109 prime outside of the used homology arms and thus show that homologous recombination has occurred at the correct location. As T7 / XZ85 primers only amplify the correct fragment from the donor plasmid source but not from *salm[1st exon-dsRed]* genomic DNA the insertion occurred by "ends-out" homologous recombination.

Table S1 All primer sequences used in this study.

Application	Primer ID	Primer name	sequence
pJET1.2-STOP-dsRed			
	XZ82	attp1-SA sense	CACACCAGGTCTCA ctcgAAGCTTCCCAGGTCAGAAG
	XZ83	attp1-SA antisense	CACACCAGGTCTCA ggtgTTAGTTAGTTAGACCTGCGG
	XZ84	sv40 sense	CACACCAGGTCTCA caccGCGATCCAGACATGAT
	XZ85	sv40 antisense	CACACCAGGTCTCA gccaGACTAGTTGATCATA ATCAGCCA
	XZ86	3p3-DsRed-SV40 sense	CACACCAGGTCTCA tggcTCGCCCGGGGATCTAA
	XZ87	3p3-DsRed-SV40 antisense	CACACCAGGTCTCA tattTCACACCGCATATGCC
	XZ88	attP2 sense	CACACCAGGTCTCA aataTTCAACCCCTTGTGTCATGTCGG
	XZ89	attP2 antisense	CACACCAGGTCTCA ttccGCGGCCCAGGTCAGAAG
	XZ195	STOP-dsRed sense	ACGTCTCA ccagTCTCGAAGCTTCCCAGGTC
	XZ196	STOP-dsRed antisense	ACGTCTCA aacaGTCTCTCGTTCCGCGGC
salm exon 1			
	XZ142	exon 1 left arm fragment 1 sense	CACACCACGTCTCA ggacGGGAGCACCATACACA
	XZ143	exon 1 left arm fragment 1 antisense	CACACCACGTCTCA atagACGTTTATGAAACTTGTTCGG
	XZ144	exon 1 left arm fragment 2 sense	CACACCACGTCTCA ctatCAGGTAAAAACAGCGACTGC
	XZ145	exon 1 left arm fragment 2 antisense	CACACCACGTCTCA ctggCCTTTGCGCGTGTTTA
	XZ148	exon 1 right arm sense	CACACCACGTCTCA tgttAGGCAATTTAATACAAATTCAAA
	XZ149	exon 1 right arm antisense	CACACCACGTCTCA agaaTTGGCTTTGCACTGAC
salm intron 1			
	XZ152	intron 1 left arm sense	CACACCACGTCTCA ggacGACTTCTGGCGGCTGT
	XZ153	intron 1 left arm antisense	CACACCACGTCTCA ctggCCTCTGATGCCACAAT

	XZ154	intron 1 right arm sense	CACACCACGTCTCA tgttGGACATATCTATACATTTAAACCAT
	XZ155	intron 1 right arm antisense	CACACCACGTCTCA gcatTAAGTTATGCGAGCGC
salm exon 3			
		Oligo Name	Sequence
	XZ157	exon 3 left arm sense	CACACCACGTCTCA cggaCTGCGGCGAGGACTACG
	XZ158	exon 3 left arm antisense	CACACCACGTCTCA ctggAAACCCCCCAATAAATTCAG
	XZ159	exon 3 right arm sense	CACACCACGTCTCA tgttGCCCCTAATGACCATCTT
	XZ160	exon 3 right arm antisense	CACACCACGTCTCA gcacATTCCAAATAGATTATTAACGTG
bent exon 11	wk049	*bent* left arm sense	AACGTCTCG tgatCTAGCCGTCAAATAGGTCTTCGG
	wk050	*bent* left arm antisense	AGCGTCTCT gcttGGTGGGCATATACGCACTC
	wk051	*bent* right arm sense	TGCGTCTCA aacgGCACGTTCCTGCACTTCTTCG
	wk052	*bent* right arm antisense	GTCGTCTCT acacGGTATTTGGCGGAAGAGCAGC
pBS-backbone			
	XZ150	pBS-GGAC-TTCT sense	CACACCACGTCTCA ttctGCAGGTGGAGCTCCAGCTTT
	XZ151	pBS-GGAC-TTCT antisense	CACACCACGTCTCA gtccGTACCCAATTCGCCCT
	XZ156	pBS-GGAC-ATGC sense	CACACCACGTCTCA atgcAGGTGGAGCTCCAGCTTT
	XZ151	pBS-GGAC-ATGC antisense	CACACCACGTCTCA gtccGTACCCAATTCGCCCT
	XZ161	pBS-CGGA-GTGC sense	CACACCACGTCTCA gtgcAGGTGGAGCTCCAGCTTT
	XZ162	pBS-CGGA-GTGC antisentse	CACACCACGTCTCA tccgTACCCAATTCGCCCT
salm sgRNAs			
	sgRNA1	gene-specific targeting oligo	TAATACGACTCACTATAG TGGGAAACGCGTAGTACCGC GTTTTAGAGCTAGAAATAGC
	sgRNA2	gene-specific targeting oligo	TAATACGACTCACTATAG GCACTTTTGTGTTTTGCCGT GTTTTAGAGCTAGAAATAGC

	sgRNA3	gene-specific targeting oligo	TAATACGACTCACTATAG AGAGGCAGAAATCGTAG GTTTTAGAGCTAGAAATAGC
	sgRNA4	gene-specific targeting oligo	TAATACGACTCACTATAG CAGTTTTCCCCCCGATTATA GTTTTAGAGCTAGAAATAGC
	sgRNA5	gene-specific targeting oligo	TAATACGACTCACTATAG CAAACGTTAACAGCTTCTAT GTTTTAGAGCTAGAAATAGC
	sgRNA6	gene-specific targeting oligo	TAATACGACTCACTATAG CAAGATCGCAAAAGGGGCGC GTTTTAGAGCTAGAAATAGC
	sgRNA7	gene-specific targeting oligo	TAATACGACTCACTATAG TTTATTGGGGGGTTTTCTAA GTTTTAGAGCTAGAAATAGC
	sgRNA8	gene-specific targeting oligo	TAATACGACTCACTATAG ATTTAAACCAGAAAACTGAT GTTTTAGAGCTAGAAATAGC
	sgRNA9	gene-specific targeting oligo	TAATACGACTCACTATAG AAGATGGTCATTAGGGGCAT GTTTTAGAGCTAGAAATAGC
	sgRNA10	gene-specific targeting oligo	TAATACGACTCACTATAG TATTTAATAAGATGGTCATT GTTTTAGAGCTAGAAATAGC
	sgRNA11	gene-specific targeting oligo	TAATACGACTCACTATAG CAAGTTTTAGAGCGAAATGA GTTTTAGAGCTAGAAATAGC
	sgRNA12	gene-specific targeting oligo	TAATACGACTCACTATAG CACGTACACCCATACTAAGG GTTTTAGAGCTAGAAATAGC
bent sgRNAs	sg_1	gene-specific targeting oligo	TAATACGACTCACTATAG ACCAGTCGTTCCTGTTATAA GTTTTAGAGCTAGAAATAGC
	sg_3	gene-specific targeting oligo	TAATACGACTCACTATAG ACAATTATCGATTAATCACT GTTTTAGAGCTAGAAATAGC
	XZ100	scaffold oligo	AAAAGCACCGACTCGGTGCCACTTTTTCAAGTTGATAACGGACTAGCCTTATTTTAACTTGCTATTTCTAGCTCTAAAAC
	XZ101	antisense primer for sgRNA production	AAAAGCACCGACTCGGTGCC
attB exchange plasmid			

	FRT-2xTY1-FRT-V5	IDT gBlock sequence	GGATCCGGAAGTTCCTATTCCGAAGTTCCTATTCTCTAGAAAGTATAGGAACTTCGAGGTCCACACTAATCAAGACCCCCTGGATGCCGAGGTGCACACCAACCAGGACCCTCTGGACcgGAAGTTCCTATTCCGAAGTTCCTATTCTCTAGAAAGTATAGGAACTTCgGGCAAGCCCATCCCCAACCCCCTGCTGGGCCTGGATAGCACCAGGATCC
	2xTY1-V5	IDT gBlock sequence	GGATCCGGAGGTCCACACTAATCAAGACCCCCTGGATGCCGAGGTGCACACCAACCAGGACCCTCTGGACGGAGGTTCCGGTGGAAGCGGAGGTAGCGGCGGATCGGGCAAGCCCATCCCCAACCCCCTGCTGGGCCTGGATAGCACCAGGATCC
pDCC6-gRNAs_cloning oligos			
	XZ123	sgRNA1 sense oligo	CTTCGTGGGAAACGCGTAGTACCGC
	XZ124	sgRNA1 antisense oligo	AAACGCGGTACTACGCGTTTCCCA
	XZ125	sgRNA3 sense oligo	CTTCGCAGAGGCAGAAATCGTAGGT
	XZ126	sgRNA3 antisense oligo	AAACACCTACGATTTCTGCCTCTG
	XZ129	sgRNA7 sense oligo	CTTCGTTTATTGGGGGGTTTTCTAA
	XZ130	sgRNA7 antisense oligo	AAACTTAGAAAACCCCCCAATAAA

	XZ131	sgRNA9 sense oligo	CTTCGAAGATGGTCATTAGGGGCAT
	XZ132	sgRNA9 antisense oligo	AAACATGCCCCTAATGACCATCTT
HDR verification			
	XZ109		AGCGAGTGTGTGGCATAATTG
	XZ120		CCACAAGGCCCTGAAGCTGA
	XZ144		CAGGTAAAAACAGCGACTGC
