# Supplementary material for: A Versatile Two-Step CRISPR- and RMCE-Based Strategy for Efficient Genome Engineering in Drosophila
Source: G3 (Bethesda). 2014 Oct 15;4(12):2409–18. doi: 10.1534/g3.114.013979 (PMC4267936; doi:10.1534/g3.114.013979)
Supplement: Supporting Information [file supp_g3.114.013979_FigureS1.pdf]

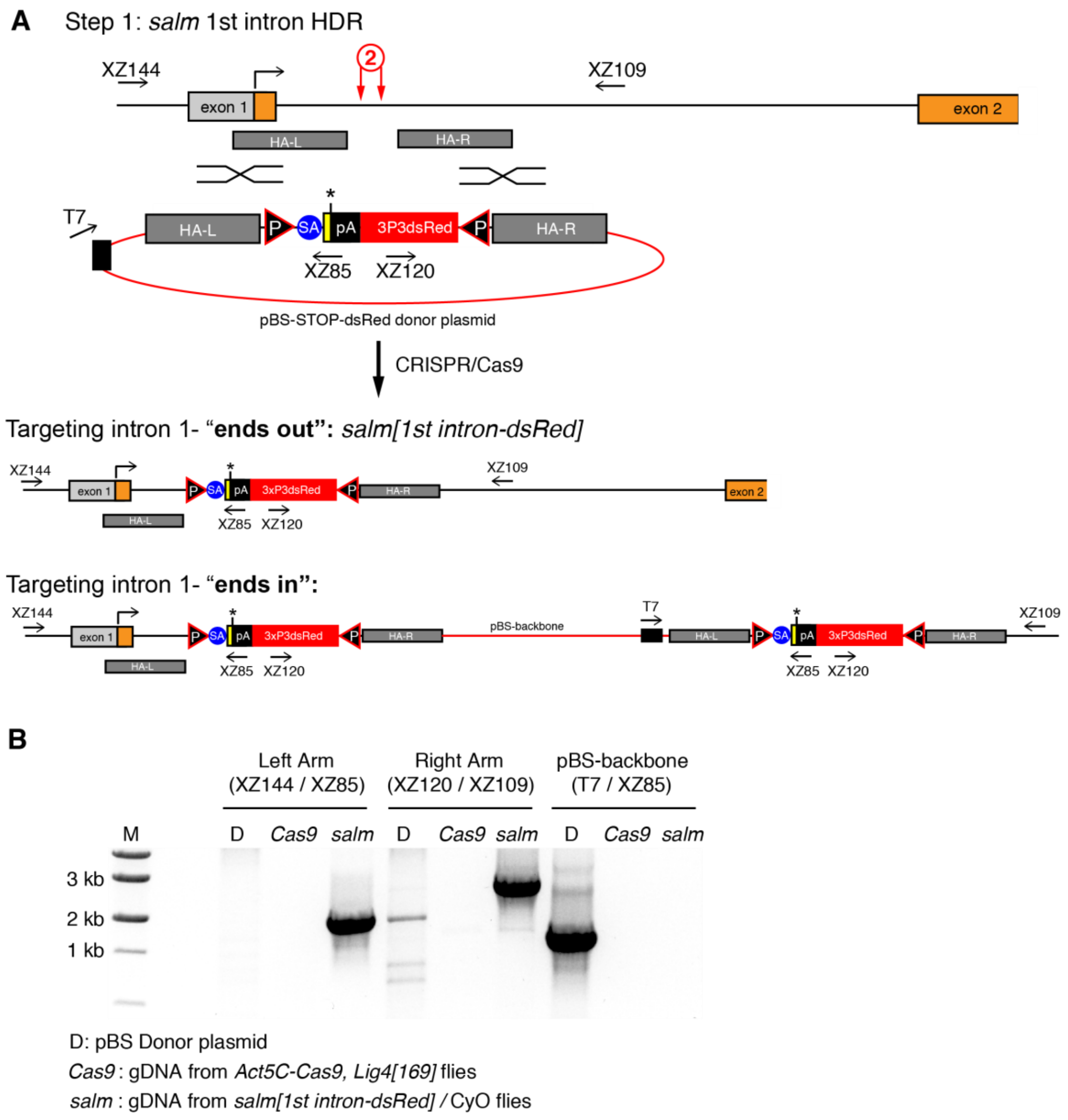

Figure S1 PCR scheme to verify the correct HDR event.

(A) Scheme for the generation of *salm[1st exon-dsRed]* by HDR. Possible results of "ends-out" and "ends-in" homologous recombination are shown, including the positions of the homology arms and the primers used for PCR. Note that only "ends-in" homologous recombination results in the pBS-backbone in the genome, which can be detected by PCR with primers T7 / XZ85. (**B**) PCR verification of the "ends-out" insertion of the dsRed-STOP cassette in the 1st intron of *salm*. Left and right arms amplify only from DNA isolated from the *salm[1st exon-dsRed]* flies. Primers XZ144 and XZ109 prime outside of the used homology arms and thus show that homologous recombination has occurred at the correct location. As T7 / XZ85 primers only amplify the correct fragment from the donor plasmid source but not from *salm[1st exon-dsRed]* genomic DNA the insertion occurred by "ends-out" homologous recombination.
